# Bibliometric and visual analysis of global research on endocrine-disrupting chemicals and children’s health: evidence, emerging concerns, and research gaps

**DOI:** 10.1080/16549716.2025.2572014

**Published:** 2025-10-16

**Authors:** Liyan Luo, Jun Guan, Shaohe Hei, Yuxin Liu, Chuyan Wu, Feng Jiang, Jiahui He

**Affiliations:** a Department of Neonatology, Dali Bai Autonomous Prefecture Maternal and Child Health Care Hospital, Dali, China; b Department of Gynecology, Obstetrics & Gynecology Hospital of Fudan University, Shanghai Key Lab of Reproduction and Development, Shanghai Key Lab of Female Reproductive Endocrine Related Diseases, Shanghai, China; c Department of Science and Education, Dali Bai Autonomous Prefecture Maternal and Child Health Care Hospital, Dali, China; d Diagnostics and Therapeutics of Intractable Diseases, Intractable Disease Research Center, Graduate School of Medicine, Juntendo University, Tokyo, Japan; e Department of Rehabilitation Medicine, The First Affiliated Hospital with Nanjing Medical University, Nanjing, China; f Department of Neonatology, Obstetrics & Gynecology Hospital of Fudan University, Shanghai Key Lab of Reproduction and Development, Shanghai Key Lab of Female Reproductive Endocrine Related Diseases, Shanghai, China

**Keywords:** Bibliometric, Web of Science, pediatric environmental health, prenatal exposure, public health policy

## Abstract

**Background:**

Endocrine-disrupting chemicals (EDCs) are environmental pollutants that interfere with hormonal systems and may pose serious risks to children’s health during critical developmental stages. Despite many studies, a comprehensive overview of global research trends is still lacking.

**Objective:**

This study aimed to provide a comprehensive bibliometric and visual analysis of global research trends on EDCs and pediatric health over the past two decades.

**Methods:**

Relevant publications from 1 January 2005 to 4 June 2025 were retrieved from the Web of Science Core Collection. Microsoft Excel, CiteSpace, VOSviewer, and the R package ‘bibliometrix’ were used to analyze publication trends, influential countries, institutions, authorship, journal distribution, keyword co-occurrence, and emerging research topics.

**Results:**

A total of 3,241 publications from 95 countries and regions were identified. The United States led with 38.29% of publications and the highest citation frequency. Among the top 10 productive authors, seven were based in the US. The Centers for Disease Control and Prevention (CDC) was the most prolific institution. Environmental Research published the largest number of articles (199, 6.14%). Major research hotspots included autism spectrum disorder, precocious puberty, child behavior, postnatal exposure, and human exposure. The most frequent keywords were ‘endocrine-disrupting chemicals,’ ‘bisphenol A,’ and ‘exposure.’

**Conclusions:**

This study provides the first systematic bibliometric overview of global research on EDCs and children’s health. The results highlight increasing academic attention and identify key contributors and emerging themes, offering guidance for future research and policy development in pediatric environmental health.

## Paper Context

- **Main findings:** This study provides the first systematic bibliometric analysis of global research on endocrine-disrupting chemicals and children’s health, identifying key contributors, collaboration networks, and emerging research hotspots.
- **Added knowledge:** The findings synthesize two decades of evidence, showing consistent links between early-life exposure and endocrine, metabolic, and neurodevelopmental outcomes, while highlighting gaps in mixture assessment and longitudinal studies.
- **Global health impact for policy and action:** The results call for stronger international collaboration, tailored strategies for high- and low-income regions, and stricter regulations to reduce harmful chemical exposures in children.

## Background

Endocrine-disrupting chemicals (EDCs) comprise various types of externally derived substances released predominantly through anthropogenic activities, which disrupt endocrine signaling and metabolic regulation, thereby impairing growth, development, and physiological homeostasis [1–3]. Despite varying definitions, EDCs share two key features: they disrupt hormone function and contribute to disease and disability across the lifespan [4]. While early research focused on receptor mimicry, more recent studies have revealed broader mechanisms, including epigenetic and enzymatic interference [2,4]. Over 1,000 EDCs have been identified, including bisphenols, phthalates, perfluoroalkyl substances (PFASs), and pesticides, which are widely used in consumer and industrial products. Due to their persistence, EDCs are detected in air, water, soil, and human tissues [2,4,5]. These chemicals can bioaccumulate and have been found in biological samples from sensitive populations, including children and pregnant women, posing potential risks throughout the life course [1,2,6].

Children are particularly vulnerable to EDCs due to their developmental stage and heightened exposure levels [4]. EDCs can cross the placenta and have been detected in fetal tissues, breast milk, and infant formula, indicating exposure from the earliest stages of life [7,8]. Infants and young children have immature metabolic and detoxification systems, thinner skin, and greater consumption of air, water, and food relative to body weight. Behaviors such as frequent hand-to-mouth behaviors further increase their exposure [2,4]. Biomonitoring studies have demonstrated higher concentrations of bisphenol A, PFASs, and other toxicants in children than in adults, with associations to obesity, metabolic dysfunction, neurodevelopmental impairment, thyroid disruption, and abnormal pubertal timing [9–11].

Although awareness of the potential harms of EDCs on children’s health has grown in recent decades, existing research remains fragmented. Most studies have examined individual compounds, specific health outcomes, or particular exposure windows, with limited integration across disciplines and insufficient attention to geographic or temporal patterns [12,13]. This has hindered the formation of a comprehensive understanding of how EDCs contribute to disease burden across developmental stages, particularly during early life. As evidence accumulates regarding the widespread presence of EDCs and their effects on multiple physiological systems, there is an urgent need to assess how the scientific community has responded globally.

Bibliometrics is an analytical approach that applies statistical techniques and mathematical modeling to assess academic literature, enabling both qualitative and quantitative evaluation of research patterns and structural characteristics [14]. By systematically analyzing elements such as countries, institutions, journals, authorship, and other key indicators, bibliometric techniques offer a comprehensive perspective on the evolution and current state of a scientific field. This method is instrumental in tracing the origins of foundational knowledge and anticipating future research directions by assessing patterns in citations, keyword co-occurrence, and publication trends. Among the widely used tools for such analyses are VOSviewer and CiteSpace, which facilitate the visualization of intellectual structures and collaborative networks. Bibliometric analysis is particularly valuable for offering a detailed and data-informed overview of scholarly activities, highlighting influential contributions and novel areas of inquiry, and supporting the development of evidence-based policies and resource allocation strategies [14]. Using bibliometric methods, we conducted a comprehensive analysis of global literature on EDCs and child health to identify research hotspots, geographic patterns, and collaboration networks, with the aim of informing future research and public health strategies to reduce early-life exposure.

## Methods

### Data sources and literature search

This study used the Web of Science Core Collection (WoSCC) as the sole data source, given its high-quality indexing, reliable citation tracking, and wide adoption in bibliometric research. Although databases such as Scopus and PubMed provide broader coverage, WoSCC was prioritized to capture the most influential studies published in leading peer-reviewed journals. To evaluate potential bias, we cross-checked the top 20 most-cited WoSCC articles against PubMed and found 19 overlaps, suggesting that the likelihood of missing key studies was low. The search strategy employed included the following terms: TS = ((‘endocrine disrupting chemical*’ OR ‘endocrine disruptor*’ OR ‘hormone disruptor*’ OR ‘environmental hormone*’) AND (‘child*’ OR ‘children’ OR ‘infant*’ OR ‘toddler*’ OR ‘pediatric’ OR ‘adolescen*’ OR ‘fetal’) AND (‘health’ OR ‘development’ OR ‘disease’ OR ‘exposure’ OR ‘neurodevelopment’ OR ‘reproductive’ OR ‘growth’ OR ‘endocrine system’)). Inclusion criteria were: (1) studies on EDCs and children’s health published between 1 January 2005 and 4 June 2025; (2) peer-reviewed original articles and reviews; and (3) articles in English. Exclusion criteria were: (1) studies not related to EDCs and children’s health, and (2) non-research materials (e.g. letters, news, editorials, proceedings, short reports, conference abstracts). Eligible records were exported in plain text format for analysis.

### Data analysis and visualization

This study performed an integrated visual bibliometric analysis utilizing VOSviewer (version 1.6.19), the R package ‘bibliometrix’ (version 4.3.1), and CiteSpace (version 6.3. R1). VOSviewer was applied to construct and visually represent bibliometric networks, including co-authorship, co-citation, and keyword co-occurrence maps. The ‘bibliometrix’ package in R provided a comprehensive suite of statistical and visualization tools, supporting the evaluation of scientific productivity and trends, as well as the identification of influential countries, institutions, and researchers. CiteSpace was employed to analyze evolving research trends and thematic shifts by generating co-citation networks and performing cluster analysis. The combined application of these three tools leveraged their respective strengths, ensuring a multidimensional, rigorous, and transparent methodological framework for examining the scientific evolution of EDC-related research in children’s health. Detailed parameter settings for VOSviewer, CiteSpace, and Bibliometrix are provided in Supplementary Table S1 for reproducibility.

## Results

### Publication outputs and temporal trends

**Figure 1.**
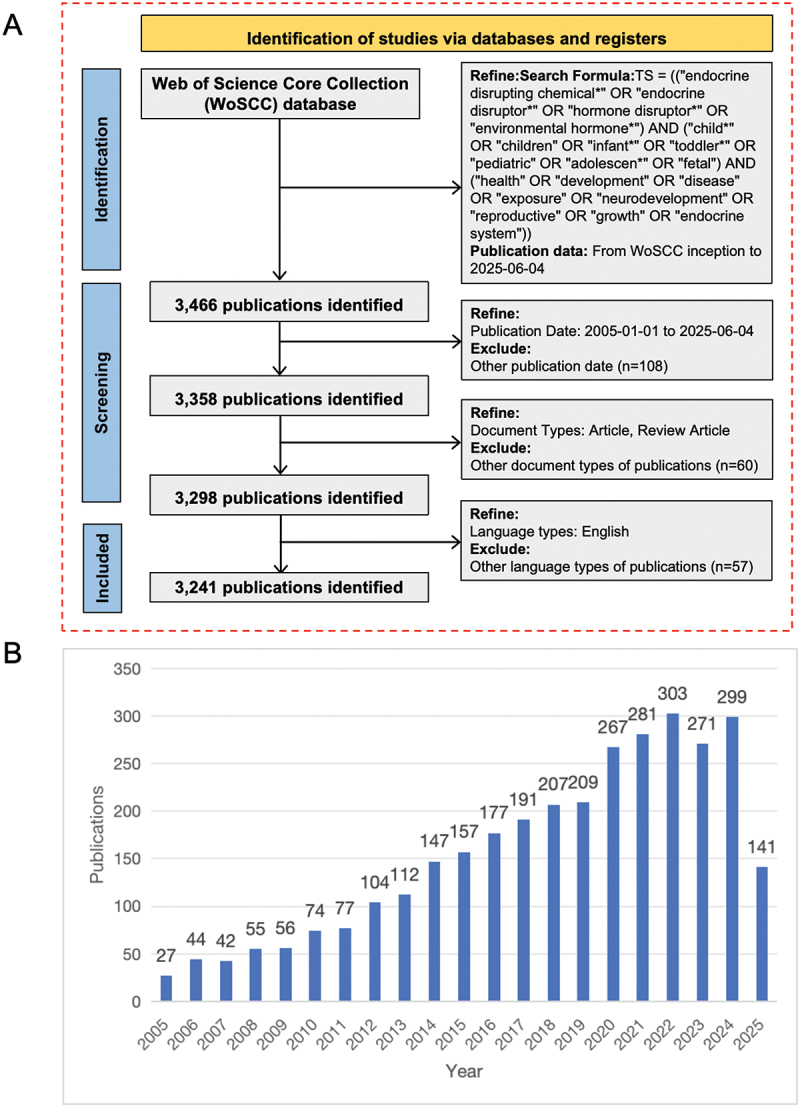
(A) Flow diagram depicting the literature retrieval and screening steps conducted in this study. (B) Worldwide publication trends related to EDCs and children’s health.

Figure 1(A) illustrates the workflow of the bibliometric analysis, detailing the selection process from the WoSCC. A total of 3,466 records were initially retrieved. After restricting the publication period to January 2005 through June 2025, 108 entries were excluded. Screening for English-language articles and reviews yielded 3,241 records for final analysis. As depicted in Figure 1(B), the number of publications related to EDCs and children’s health showed a clear upward trajectory, which can be broadly divided into three phases. The initial phase (2005–2009) had relatively low output, ranging from 27 to 56 publications per year, reflecting the early stage of scientific inquiry in this field. The second phase (2010–2017) marked steady growth, with annual publications rising from 74 to 191, indicating increasing academic interest and expanding research activity. The third phase (2018–2024) demonstrated a high-output plateau, with annual publication counts consistently exceeding 200. A peak was observed in 2022 with 303 publications, followed by a slight decline in 2023 (271) and a near-rebound in 2024 (299). As of mid-2025, 141 articles have been recorded, though the total for the year was expected to increase.

### Worldwide research cooperation and productivity

**Figure 2.**
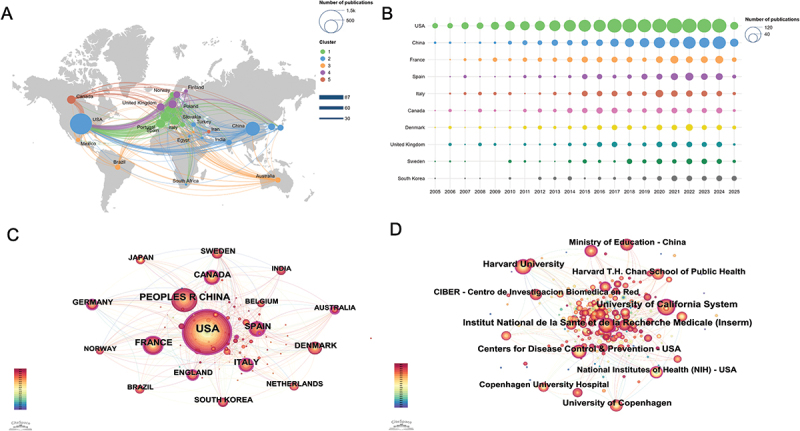
Collaboration networks in research on EDCs and children’s health. (A) Map of global collaborative relationships. (B) Publication trends of the top 10 countries by number of publications. (C) Collaboration network among countries/regions. (D) Network of institutional collaborations.

Research on EDCs and children’s health involved 95 countries and regions. Figure 2(A) displays the global collaboration network in this field. The size of each node reflects the number of publications, while the thickness of the connecting lines represents the strength of collaboration. The United States (US) had the highest publication volume and demonstrated strong international collaboration, especially with Canada, China, the United Kingdom (UK), and the European Union (EU) countries. China ranked second in output and served as a key partner in Asia. European countries, particularly Italy, Spain, and the UK, formed a dense cooperative cluster. Emerging contributors such as Brazil, India, and Australia showed moderate involvement. Overall, the network revealed concentrated collaboration across North America, Europe, Australia, and East Asia, with regional clusters linked by cross-continental partnerships. Figure 2(B) shows the annual publication trends of the top 10 most productive countries from 2005 to mid-2025. The US consistently led in output, with a notable increase after 2013 and a peak around 2021. China followed with a steady rise, particularly after 2017. European countries such as France, Spain, and Italy maintained relatively stable contributions, while Canada, the UK, and Denmark showed moderate but consistent activity. Emerging contributions from Sweden and South Korea became more visible in the past decade. Figure 2(C) illustrates the country co-occurrence network, with the US occupying the central position, reflecting its highest frequency of collaboration and pivotal role in international research efforts. China, France, and Spain also demonstrated strong co-occurrence links, indicating their active engagement in global scientific cooperation.

Altogether, 228 research institutions contributed publications on EDCs and children’s health. Figure 2(D) presents the institutional co-occurrence network, highlighting key collaboration patterns. Leading institutions with the strongest collaborative ties included the Centers for Disease Control and Prevention (CDC), the University of California System, and Institut National de la Santé et de la Recherche Médicale. Other active contributors included the University of Copenhagen, Centro de Investigación Biomédica en Red (CIBER), and Harvard T.H. Chan School of Public Health. Most of these institutions were based in the US, reinforcing its central role in global research. Tables 1 and 2 summarize the top 10 most productive countries and institutions. The US contributed 38.29% of all publications, significantly surpassing other countries. Papers published in the US received 80,729 citations, far exceeding other countries/regions, with a citation-to-publication ratio of 65.05, ranking second globally and indicating generally high research quality. The top 10 institutions contributed 23.17% of the total literature, reflecting the pivotal role of key academic centers. Among the 10 most productive institutions, eight were from the US and two from Europe (Denmark and Spain). These entities functioned as major research hubs, fostering international collaboration and advancing the scientific understanding of early-life exposure to EDCs.

**Table 1.** Top 10 countries contributing to research on EDCs and children’s health.

Rank	Country	Count	Centrality	Citation	Citation per publication
1	United States	1241(38.29%)	0.29	80,729	65.05
2	China	479 (14.77%)	0.08	14,354	29.97
3	France	265 (8.17%)	0.13	13,013	49.10
4	Spain	203 (6.26%)	0.11	11,684	57.56
5	Italy	203 (6.26%)	0.14	9862	48.58
6	Canada	194 (5.98%)	0.14	9681	49.90
7	Denmark	180 (5.55%)	0.10	12,574	69.86
8	Sweden	128 (3.94%)	0.08	6115	47.77
9	United Kingdom	126 (3.88%)	0.10	6081	48.26
10	South Korea	126 (3.88%)	0.02	3755	29.80

**Table 2.** Top 10 institutions contributing to research on EDCs and children’s health.

Rank	Institution	Country	Count	Total citations	Average citation
1	Centers for Disease Control & Prevention	United States	100 (3.08%)	8592	85.92
2	University of Copenhagen	Denmark	92 (2.83%)	6329	68.79
3	Icahn School of Medicine at Mount Sinai	United States	82 (2.53%)	3483	42.48
4	University of Michigan	United States	76 (2.34%)	4761	62.64
5	Universidad de Granada	Spain	75 (2.31%)	4402	58.69
6	Harvard T.H. Chan School of Public Health	United States	70 (2.15%)	2505	35.79
7	New York University	United States	68 (2.09%)	3739	54.99
8	Columbia University in the City of New York	United States	67 (2.06%)	3775	56.34
9	Harvard Medical School	United States	62 (1.91%)	1753	28.27
10	Brown University	United States	59 (1.82%)	3157	53.51

### Analysis of author influence and collaboration

**Figure 3.**
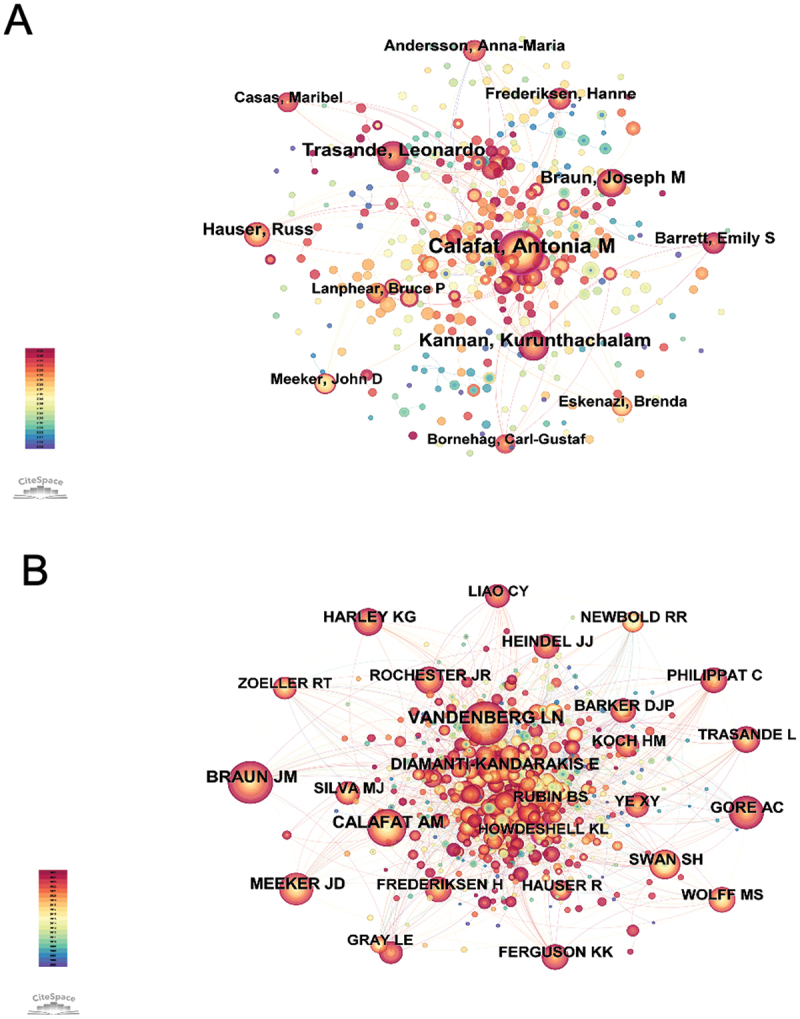
Authorship and co-citation visualization in research on EDCs and children’s health. (A) Network map of contributing authors in the field. (B) Visualization of authors frequently co-cited within this research area.

Between 2005 and 2025, 377 authors published studies on EDCs and children’s health, and 604 authors were co-cited in this field. Figure 3(A) displays the co-authorship network of leading researchers. Calafat, Antonia M. appeared as the most central contributor, with the highest co-authorship frequency. Trasande, Leonardo also demonstrated strong collaborative activity, followed by Kannan, Kurunthachalam and Hauser, Russ, who maintained consistent links with other active researchers. These authors formed the core of the collaborative network, underscoring their pivotal roles in shaping the research landscape. Figure 3(B) visualizes the author co-citation network generated using CiteSpace. Braun, Joseph M. and Vandenberg, Laura N. were the most prominently positioned nodes, reflecting their sustained and influential contributions. Calafat, Antonia M. also emerged as a highly co-cited author, indicating broad recognition across studies. Meeker, John D. and Koch, Holger M. showed frequent co-citation patterns, underscoring their relevance in EDC-related literature. Table 3 lists the 10 most prolific and frequently co-cited authors. Among the productive authors, seven were from the US. Similarly, among the top co-cited authors, eight were affiliated with US institutions. These 10 authors published a total of 462 articles, accounting for 14.25% of all publications. The top 10 co-cited authors received 6,361 citations, with an average of 636 citations each. The strong overlap between prolific and frequently cited authors underscored their central role in advancing the field.

**Table 3.** Top 10 authors and co-cited authors on EDCs and children’s health research.

Rank	Authors	Count	Country	Co-cited authors	Citations	Country
1	Calafat, Antonia M.	97(2.99%)	United States	Braun, Joseph M.	1084	United States
2	Trasande, Leonardo	56(1.72%)	United States	Vandenberg, Laura N.	1084	United States
3	Kannan, Kurunthachalam	54(1.66%)	United States	Calafat, Antonia M.	736	United States
4	Hauser, Russ	45(1.38%)	United States	Meeker, John D.	560	United States
5	Braun, Joseph M.	44(1.35%)	United States	Koch, Holger M.	514	Germany
6	Lanphear, Bruce P.	34(1.04%)	Canada	Swan, Shanna H.	507	United States
7	Frederiksen, Hanne	34(1.04%)	Denmark	Newbold, RR	503	United States
8	Meeker, John D.	33(1.01%)	United States	Transande, Leonardo	474	United States
9	Andersson, Anna-Maria	33(1.01%)	Denmark	Gore, Andrea C.	455	United States
10	Barrett, Emily S.	32(0.98%)	United States	Frederiksen, Hanne	444	Denmark

### Core journals and citation mapping

**Figure 4.**
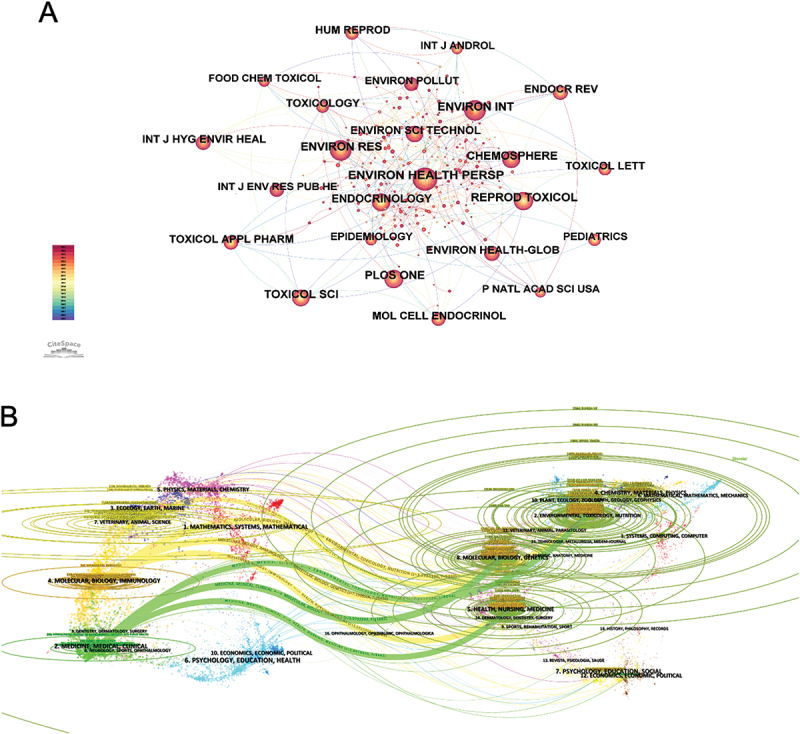
Journal co-citation visualization in research on EDCs and children’s health. (A) Network of journal co-citations within this field. (B) Dual-map overlay illustrating the disciplinary connections of journals related to EDCs and children’s health research.

Figure 4(A) displays the journal co-citation network in the field. *Environmental Health Perspectives* was the most frequently co-cited journal, occupying the central position. Other highly co-cited journals included *Environmental Science & Technology*, *Chemosphere*, *Environmental Research*, *Endocrinology*, and *Reproductive Toxicology*. The network also highlighted contributions from *Toxicology, PLoS One, Pediatrics*, and *PNAS*, reflecting the interdisciplinary nature of the field across environmental science, toxicology, public health, and pediatrics. The dual-map overlay generated by CiteSpace illustrated the disciplinary evolution of research. As shown in Figure 4(B), it visualized the citation trajectories from citing journals (on the left) to cited journals (on the right). Two main citation trajectories were identified: publications in environmental/toxicology/nutrition journals predominantly cited work in molecular/biology/genetics journals, while articles in medicine/medical/clinical journals mainly referenced health/nursing/medicine literature. This pattern suggested an active knowledge flow from biomedical sciences toward environmental and public health research concerning EDCs and children’s health. Tables 4 and 5 present the top 10 most productive journals and co-cited journals. *Environmental Research* published the largest number of articles (199, 6.14%), followed by *Environment International* (180 articles, 5.55%) and *Environmental Health Perspectives* (110 articles, 3.39%). Among these, *Environment International* had the highest impact factor (IF = 10.3) and, along with the others, was classified within Q1 or Q2 quartiles. Co-citation frequency serves as an indicator of a journal’s scientific influence. *Environmental Health Perspectives* was the most frequently co-cited journal (18,191 times), followed by *Environment International* (7,098) and *Environmental Research* (6,008). Notably, *Environment International* was cited 4,080 times and held the highest IF (10.9) among the top 10 co-cited journals. All journals in the co-citation list were also within Q1 or Q2 categories, reflecting their strong academic influence.

**Table 4.** Top 10 journals on EDCs and children’s health research.

Rank	Journal	Count	IF	JCR
1	Environmental Research	199 (6.14%)	7.7	Q1
2	Environment International	180 (5.55%)	10.3	Q1
3	Environmental Health Perspectives	110 (3.39%)	10.1	Q1
4	Science of the Total Environment	108 (3.33%)	8.2	Q1
5	Reproductive Toxicology	88 (2.71%)	3.3	Q2
6	Chemosphere	77 (2.37%)	8.1	Q1
7	International Journal of Environmental Research and Public Health	69 (2.12%)	4.6	Q2
8	Environmental Pollution	66 (2.03%)	7.6	Q1
9	International Journal of Hygiene and Environmental Health	65 (2.00%)	4.5	Q1
10	PLOS ONE	57 (1.75%)	2.9	Q1

**Table 5.** Top 10 co-cited journals on EDCs and children’s health research.

Rank	Co-cited Journal	Citations	IF	JCR
1	Environmental Health Perspectives	18,191	10.1	Q1
2	Environment International	7098	10.3	Q1
3	Environmental Research	6008	7.7	Q1
4	Reproductive Toxicology	4957	3.3	Q2
5	Endocrinology	4534	3.8	Q2
6	Environmental Science &Technology	4080	10.9	Q1
7	Toxicological Sciences	4007	3.4	Q2
8	Chemosphere	3909	8.1	Q1
9	Science of the Total Environment	3786	8.2	Q1
10	PLOS ONE	3448	2.9	Q1

### Highly cited references and foundational studies

**Figure 5.**
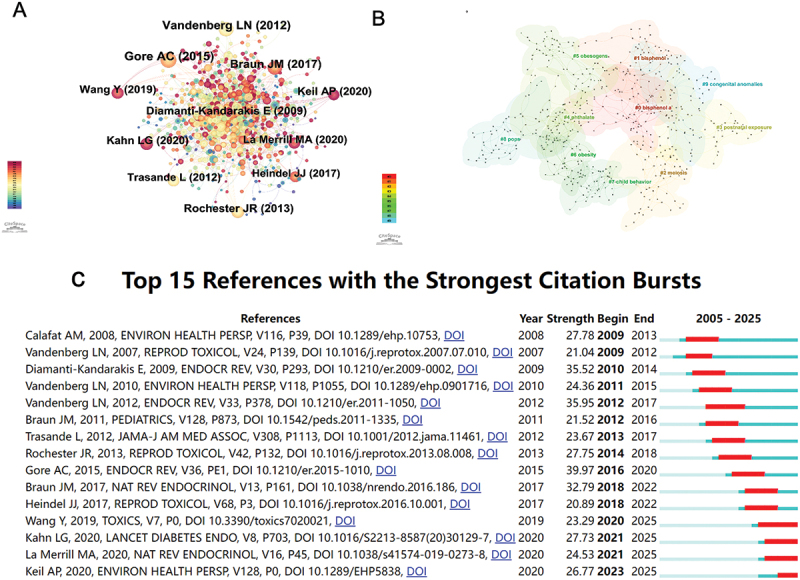
Visualization of co-cited references in research on EDCs and children’s health. (A) Network of co-cited references within the field. (B) Cluster map of co-cited references in this area. (C) Top 15 references exhibiting the strongest citation bursts.

Reference co-citation analysis, a key function of CiteSpace, is frequently applied to uncover major research themes within a given field. Figure 5(A) displays the resulting co-citation network of references. Each node represents a highly cited reference, with node size corresponding to its co-citation frequency. Key publications such as Vandenberg LN (2012), Gore AC (2015), Braun. JM (2017) were positioned centrally, followed by Diamanti-Kandarakis E (2009) and Trasande L (2012), reflecting their foundational roles in shaping the discourse on EDCs and children’s health. The reference co-citation clustering map (Figure 5(B)) identified 10 major clusters, revealing the thematic structure of the field. The largest clusters were bisphenol A (#0) and bisphenol (#1), confirming their central role across multiple health domains. Clusters such as phthalate (#4), obesogens (#5) and obesity (#6) reflected the strong focus on metabolic disruptions. The appearance of meiosis (#2) and congenital anomalies (#9) suggested increasing attention to reproductive and developmental toxicity. Meanwhile, clusters on postnatal exposure (#3) and child behavior (#7) pointed to the growing interest in neurodevelopmental effects. Citation burst analysis identified the 15 references with the most prominent citation surges, as illustrated in Figure 5(C), indicating influential publications that gained rapid academic attention during specific time spans. The strongest burst was observed for Gore et al. (2015) (strength = 39.97), followed by Vandenberg et al. (2012) and Diamanti-Kandarakis et al. (2009), which highlighted the importance of mechanisms, low-dose effects, and endocrine disruption pathways. More recent bursts from Wang et al. (2019), Kahn et al. (2020), and Keil et al. (2020) suggested a shifting focus toward new pollutants, metabolic programming, and advanced analytical methodologies. Table 6 summarizes the top 10 co-cited references on EDCs and children’s research. The article entitled ‘*Endocrine-Disrupting Chemicals: An Endocrine Society Scientific Statement*’ in *Endocrine Reviews* (IF = 22.0) was the most co-cited reference, with Evanthia Diamanti-Kandarakis serving as lead author.

**Table 6.** Top 10 co-cited references on EDCs and children’s health research.

Rank	Co-cited reference	Citations
1	Diamanti-kandarakis e, 2009, endocr rev, v30,p293	382
2	Vandenberg ln, 2012, endocr rev, v33, p378	286
3	Gore ac, 2015, endocr rev, v36, pe1	270
4	Vandenberg ln, 2007, reprod toxicol, v24, p139	248
5	Calafat am, 2008, environ health persp, v116, p39	230
6	Swan sh, 2005, environ health persp, v113, p1056	206
7	Braun jm, 2017, nat rev endocrinol, v13, p161	168
8	Hornung r.w., 1990, appl. occup. environ. hyg,v5, p46	162
9	Skakkebeek ne, 2001, hum reprod, v16, p972	158
10	Schönfelder g, 2002, environ health persp, v110, pa703	155

### Keyword co-occurrence and research hotspots

**Figure 6.**
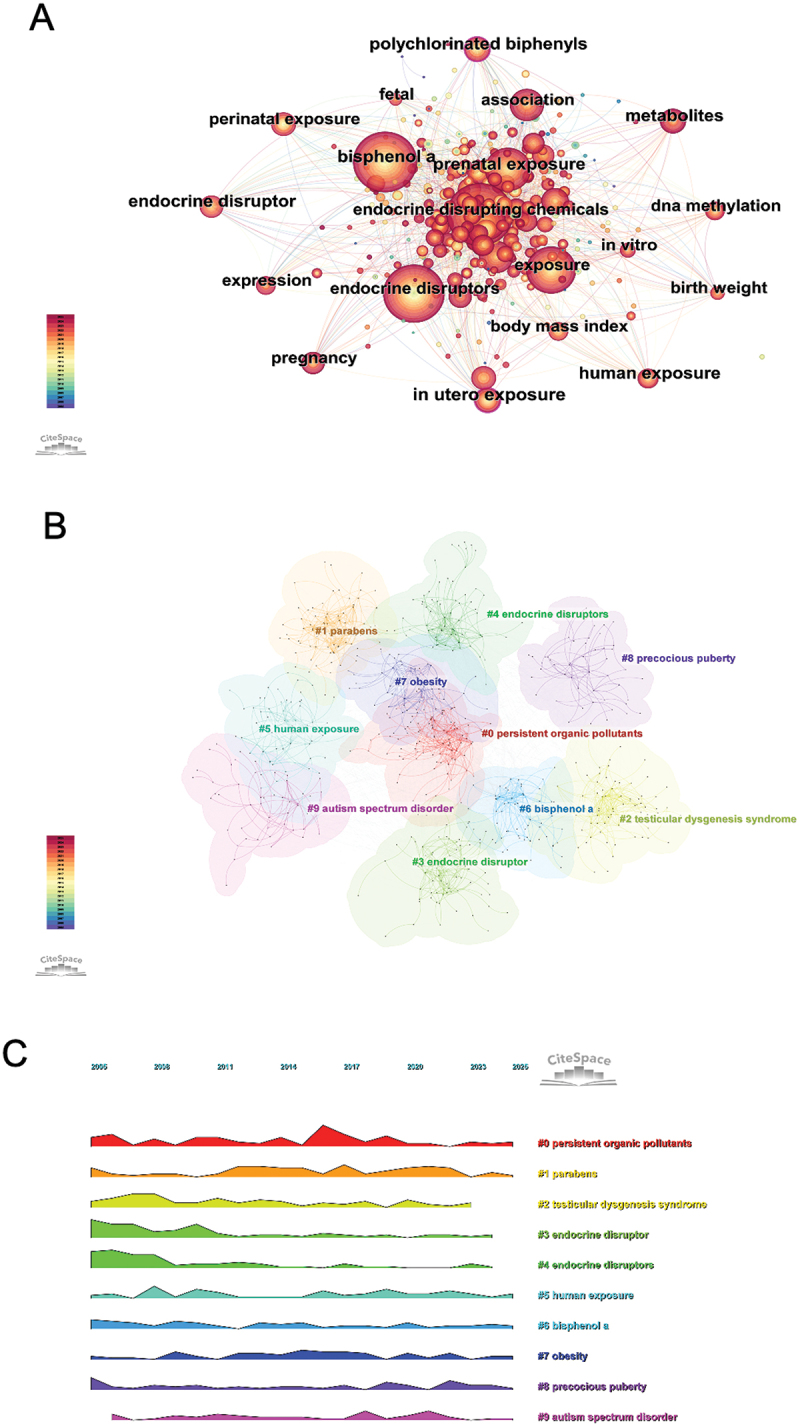
Keyword analysis in EDCs and children’s health research. (A) Network visualization of keyword co-occurrence. (B) Clustered map of keyword groupings. (C) Thematic landscape of keywords in the research field.

Analyzing keywords offers an efficient means of capturing the thematic landscape and developmental trajectory of a research field. Figure 6(A) shows the co-occurrence network of keywords related to EDCs and children’s health. Frequently occurring terms included ‘endocrine disrupting chemicals,’ ‘bisphenol A,’ ‘prenatal exposure,’ ‘birth weight,’ and ‘in utero exposure,’ reflecting research focus on early-life exposure and developmental outcomes. Keywords such as ‘phthalates,’ ‘DNA methylation,’ and ‘metabolites’ suggested growing interest in specific compounds and molecular mechanisms. Figure 6(B) presents the keyword clustering analysis, revealing 10 major thematic clusters. Cluster #0 (persistent organic pollutants) reflected the core focus on long-lasting environmental toxins. Clusters #1 (parabens), #6 (bisphenol A), and #4/#3 (endocrine disruptors/disruptor) highlighted the centrality of specific EDCs and their classification. Notably, clusters #2 (testicular dysgenesis syndrome), #7 (obesity), #8 (precocious puberty), and #9 (autism spectrum disorder) indicated growing interest in reproductive, metabolic, and neurodevelopmental outcomes. Cluster #5 (human exposure) emphasized real-world monitoring and exposure assessment as a foundational research trend. Figure 6(C) illustrates the temporal evolution of these clusters. Persistent organic pollutants (#0) maintained consistent prominence, peaking around 2017. In contrast, clusters like bisphenol A (#6), obesity (#7), and human exposure (#5) showed a gradual rise in recent years, reflecting sustained or emerging research interest. Meanwhile, neurodevelopment-related themes like autism spectrum disorder (#9) and precocious puberty (#8) received modest but increasing attention. These temporal trends underscored a broadening scope of research, with a shift from chemical identification toward long-term health effects and exposure pathways.

**Figure 7.**
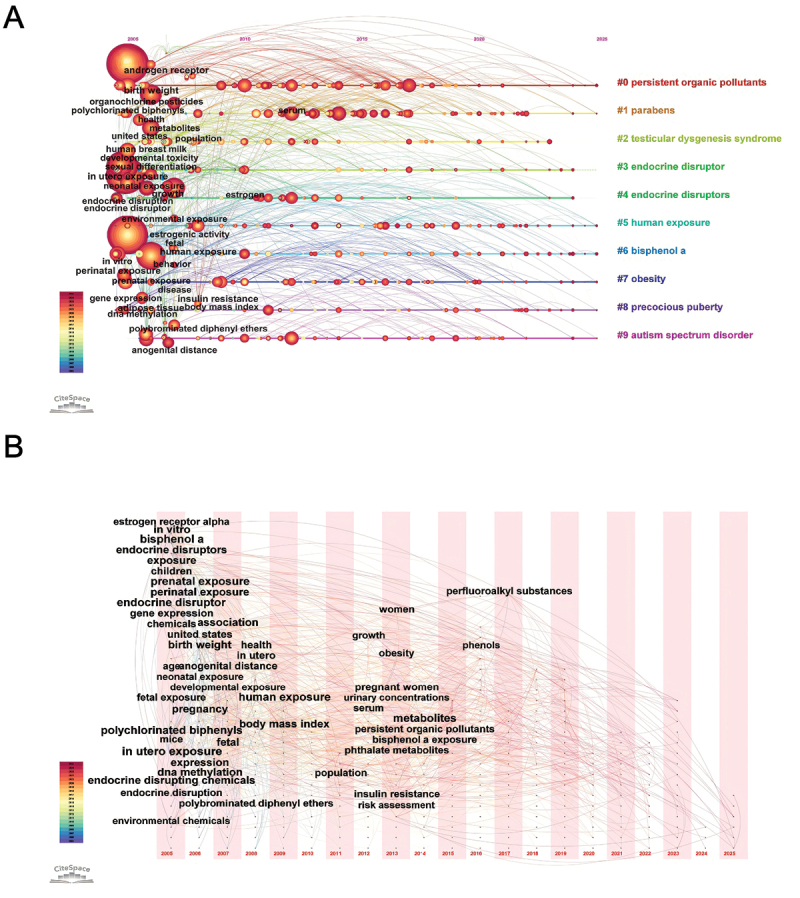
Temporal keyword analysis in EDCs and children’s health research. (A) Timeline visualization of keyword clusters related to the topic. (B) Timezone mapping of keyword emergence and evolution within the field.

**Figure 8.**
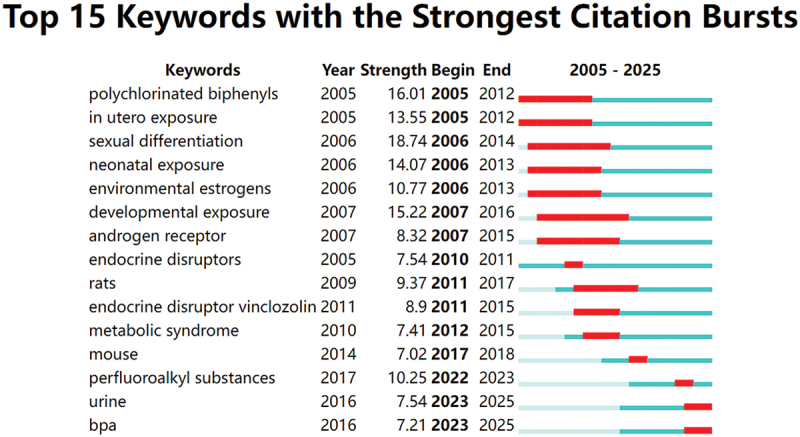
Top 15 keywords exhibiting the most significant citation bursts. Blue bars denote intervals of lower citation activity, whereas red bars highlight phases of heightened scholarly attention.

Figure 7(A) presents the timeline of keyword clusters, highlighting shifts in research themes on EDCs and child health over the 2005–2025 period. Cluster #0 (persistent organic pollutants) remained an enduring focus, reflecting its foundational role in EDC studies. In contrast, clusters such as #8 (precocious puberty) and #9 (autism spectrum disorder) gained prominence in recent years, signaling a shift toward neurodevelopmental and endocrine-related outcomes. The timezone visualization (Figure 7(B)) provided further insight into the evolution of research priorities. Early studies emphasized exposure pathways and developmental effects, with keywords like ‘in utero exposure,’ ‘birth weight,’ and ‘endocrine disruptors’ dominating the landscape. In later years, research shifted toward specific compounds and biomonitoring, as emerging terms such as ‘phenols,’ ‘serum,’ and ‘perfluoroalkyl substances’ indicated increased attention to internal exposure assessment and novel contaminants. Figure 8 presents the 15 keywords with the most significant citation bursts, with red bars denoting periods of intensified academic focus. Early bursts (‘polychlorinated biphenyls’ and ‘sexual differentiation’) reflected foundational toxicological concerns, whereas recent bursts (‘urine,’ ‘BPA,’ and ‘perfluoroalkyl substances’) highlighted a growing emphasis on human biomonitoring and exposure quantification. Together, these findings underscored the thematic transition from chemical identification and general exposure assessment toward specific health effects and measurable biological indicators.

## Discussion

### Global research landscape

Research on EDCs and children’s health grew steadily over the past two decades and reached a peak in 2022, reflecting increased public and scientific concern about environmental exposures during early life. Key initiatives such as the comprehensive reports from the World Health Organization (WHO) and United Nations Environment Programme (UNEP) in 2012 highlighted the vulnerability of children to EDC exposure and called for urgent research and policy action [15]. Globally, research was conducted in 95 countries/regions, with the US, China, France, Spain, and Italy leading output. The US alone accounted for 38.2% of total publications and achieved the highest citation count (80,729), reflecting both volume and impact. This leadership is supported by substantial funding, prominent institutions, broader regulatory and societal drivers. The 2016 revision of the Toxic Substances Control Act (TSCA) introduced more stringent chemical safety requirements, providing strong policy momentum for research [16]. Long-term federal investments, particularly through the National Institute of Environmental Health Sciences (NIEHS) Children’s Environmental Health Research Centers, enabled large-scale cohort studies that generated influential findings [17]. At the same time, advocacy groups such as the Environmental Working Group (EWG) elevated public awareness and media attention, indirectly shaping research priorities and funding decisions [18]. Together, these intersecting forces help explain the US’ dominant position in this field. By contrast, China showed rapid growth but had a lower citation-to-publication ratio (29.97), underscoring the need to improve research quality and strengthen international collaboration. Notably, eight of the ten most productive institutions were based in the US, and collaboration patterns revealed a preference for intra-national networks, highlighting the importance of fostering stronger cross-border cooperation.

### Key contributors and citation patterns

Among the top 10 most productive authors who each published at least 32 articles, seven were based with institutions in the US, reflecting the country’s leading role in this field. Calafat, Antonia M. ranked first with 97 publications, followed by Trasande, Leonardo (56) and Kannan, Kurunthachalam (54). Co-citation analysis revealed that Braun, Joseph M. and Vandenberg, Laura N. were the most influential authors, each with 1,084 citations, underscoring their foundational impact. Braun investigated how early-life exposure to EDCs such as BPA and phthalates may be associated with increased risks of neurobehavioral disorders and childhood obesity [19], while Vandenberg et al. revealed that flawed exposure assessments may underestimate chemical risks, calling for improved methods to protect public health [20]. Antonia M. Calafat, the most prolific and highly cited author (736 citations), validated the indirect method for BPA biomonitoring [21].

The top 10 journals collectively published 1,019 articles, representing 31.44% of the total output in this field. Notable outlets such as *Environmental Research*, *Environment International*, and *Environmental Health Perspectives* demonstrated a concentrated interest in topics related to EDCs and child health, offering valuable publication venues for researchers. Furthermore, author and co-citation analyses identified Antonia M. Calafat, Joseph M. Braun, and Leonardo, Trasande as prominent figures across all three bibliometric indicators, underscoring their substantial influence and potential as collaborative partners. The scholarly impact of journals was reflected in their co-citation frequency, serving as a metric of their relevance and visibility within the academic community [22]. The journal with the highest number of co-citations was *Environmental Health Perspectives* (18191), followed by *Environment International* (7098) and *Environmental Research* (6008). All co-cited journals were classified within the Q1 or Q2 quartiles.

Among the top 10 most co-cited references, the most frequently cited was the article ‘*Endocrine-Disrupting Chemicals: An Endocrine Society Scientific Statement*’ published in *Endocrine Reviews* (IF = 22.0), with 382 citations. The statement highlighted that EDCs interfere with multiple hormonal systems and pose significant public health concerns [23]. Several highly cited studies focused on BPA exposure. Schönfelder et al. first confirmed the accumulation of parent BPA in the maternal-fetal-placental unit [24]. Calafat et al. provided the first US nationally representative data, revealing widespread BPA detection in children and adults [25]. Vandenberg et al. further pointed out that typical concentrations in human fluids may exceed thresholds that trigger molecular changes in vitro or in animal models [26]. Building on these findings, Braun emphasized links between early-life EDC exposure and childhood neurobehavioral disorders and obesity, stressing children’s vulnerability and the need for better exposure assessment to inform prevention [19].

### Emerging hotspots and thematic trends

Recognizing research hotspots is essential for understanding the field’s development. Based on co-occurrence and temporal clustering, endocrine disruptors (cluster #3/4), bisphenol A (cluster#6), and testicular dysgenesis syndrome (cluster #2) were identified as early research hotspots. Parabens (#1), obesity (cluster#7), and persistent organic pollutants (cluster #0) represented mid-term hotspots, reflecting a growing focus on early-life biomonitoring. Recent hotspots included autism spectrum disorder (cluster #9), precocious puberty (cluster #8) and Human exposure (cluster#5), highlighting increasing concern over neurodevelopmental and endocrine impacts. Citation burst analysis (Figure 8) further emphasized keywords like urine, BPA, and PFAS, indicating growing interest in exposure pathways and health outcomes during childhood. These temporal patterns likely reflect increasing scientific attention, methodological advances, and growing public health concern regarding early-life exposures and neuroendocrine outcomes

### Hotspot 1: exposure pathways and biomonitoring in early life

Early-life exposure to EDCs occurs through multiple pathways, including prenatal transfer, medical interventions, diet, and consumer products. Prenatal exposure arises through placental transfer, primarily via passive diffusion due to the lipophilic nature of many EDCs, directly linking maternal exposure to fetal burden [27]. Persistent organic pollutants (POPs) and other lipophilic EDCs cross the maternal – fetal interface and can be measured in cord blood and placenta, supporting evidence of fetal accumulation during sensitive developmental windows [28]. Medical interventions represent another important source. Devices used in neonatal intensive care units (NICUs) release phthalates, BPA, parabens, and PFAS, with preterm and low-birth-weight infants being especially vulnerable [29]. Intensive care procedures can result in substantial phthalate exposure, particularly from Di(2-ethylhexyl) phthalate (DEHP), providing a route of acute, high-dose exposure in these clinical settings [30]. Dietary intake is the dominant exposure route in early life. Breast milk, infant formula, and food packaging contain phytoestrogens, POPs, PFAS, and plastic-related chemicals [6,31]. Packaging materials such as plastic containers, films, cans, and greaseproof paper further contribute to exposure [6]. Additional risks arise from personal care products and cosmetics, including lotions, powders, and shampoos, which have been associated with elevated urinary phthalate metabolites in infants [32]. Clothing also contributes, as residues such as flame retardants and plasticizers can migrate via skin contact or inhalation of semi-volatile organic compounds (SVOCs) [31].

Biomonitoring approaches vary by matrix. Breast milk is a widely used non-invasive indicator that reflects both maternal and infant exposure [33]. Despite contamination concerns, breastfeeding remains beneficial and is still recommended. Urine is particularly suitable for infants because it is non-invasive and feasible for repeated sampling [31]. Recent biomarker and multi-omics studies further demonstrated that early-life EDC exposure is linked to disruptions in metabolic and neuroendocrine pathways, including serotonin, leptin, and tryptophan – kynurenine metabolism, offering mechanistic insights into downstream biological effects [34]. Bisphenol analogues (BPA, BPF, BPS) are commonly detected in children, with trends reflecting regulatory restrictions. Higher levels have been associated with lower socioeconomic status and exposure to secondhand smoke [35]. Blood sampling, though limited in infants due to ethical and practical challenges, provides valuable information in older children, including markers linked to neurodevelopmental and metabolic outcomes such as serotonin, leptin, and kynurenine [34]. Saliva and perinatal matrices also provide alternative sources, though each has limitations. Saliva collection in infants carries contamination risks, while perinatal matrices, though informative, are logistically challenging [31]. The placenta, rich in hormone receptors, is particularly susceptible to EDC accumulation and may serve as a long-term indicator of in utero exposure [36]. Collectively, these pathways highlight the complexity of early-life EDC exposure and the need for biomonitoring across multiple sources. Evidence consistently indicates that dietary intake and consumer products are the main contributors, with urine and breast milk serving as the most practical matrices for assessing cumulative exposure in infants.

### Hotspot 2: EDCs and endocrine developmental outcomes

EDCs are associated with a wide range of endocrine disorders, particularly those involving reproductive and metabolic systems. Experimental evidence indicated that compounds such as PCBs, BPA, and phthalates can cross the blood – brain barrier, induce hypothalamic inflammation, and disrupt the gonadotropic axis, ultimately impairing reproductive function [37]. Many EDCs also act directly on nuclear receptors including estrogen receptors, androgen receptors, and PPARγ, altering transcriptional programs that regulate adipogenesis, gonadal differentiation, and steroidogenesis. These receptor-mediated pathways, together with indirect mechanisms such as hypothalamic inflammation triggered by halogenated compounds and plasticizers, plausibly explain shifts in pubertal timing and reductions in ovarian reserve markers [38]. Epidemiological findings supported these mechanistic insights. Data from large birth cohorts, such as the INMA study in Spain, showed that prenatal exposure to persistent chemicals including DDE, HCB, and PCBs was associated with accelerated BMI gain in childhood, which may predispose to long-term metabolic risk [39]. A meta-analysis further demonstrated that prenatal exposure to organochlorines and PFAS was negatively associated with neonatal TT4 levels, indicating disruption of fetal thyroid function during critical developmental windows [40]. Similarly, bisphenol exposure has been linked to altered thyroid hormone levels in female offspring, with clear dose-dependent effects and sex-specific sensitivity [41].

Additional evidence suggested that EDCs compromise ovarian reserve development and disrupt the hypothalamic – pituitary – ovarian axis, thereby increasing the risk of reproductive disorders such as early menopause and polycystic ovary syndrome [42]. Mechanistic studies, including network toxicology analyses, have identified oxidative stress, apoptosis, and estrogen signaling as key mediators of phthalate toxicity, with core targets such as PTGS2, CASP3, and ESR1 linking molecular perturbations to tissue-level endocrine dysfunction [43,44]. Converging evidence demonstrated that EDCs disrupt thyroid, reproductive, and metabolic pathways, with outcomes including accelerated childhood growth, altered pubertal timing, and increased risk of reproductive disorders.

### Hotspot 3: neurodevelopmental and behavioral disorders

Neurodevelopmental disorders have become a major research focus, with a growing number of studies addressing autism spectrum disorder (ASD). This trend is driven by several factors: the rising prevalence of ASD, increasing recognition of environmental contributions beyond diagnostic expansion, and methodological advances such as longitudinal cohort designs, biomarker-based exposure assessment, and epigenetic profiling, which together improved the ability to detect associations that were previously difficult to capture [1,11,45]. Epidemiological studies supported links between prenatal or early-life exposure to EDCs and adverse neurodevelopment. The Swedish SELMA cohort reported that prenatal exposure to a mixture of 15 EDCs was associated with language delay at 30 months, underscoring the importance of assessing real-world chemical mixtures [1]. Similarly, findings from the MARBLES cohort indicated that higher urinary concentrations of phthalate metabolites in infancy predicted lower cognitive scores at 36 months, even without clinical diagnoses [46]. Mechanistic research provided biological plausibility for these associations. Thyroid hormones and sex steroids regulate neuronal proliferation, migration, myelination, and synaptogenesis; disruption of these pathways by EDC-induced maternal thyroid insufficiency or altered sex steroid signaling during critical prenatal periods may impair brain development [11,47]. Prenatal EDC exposure has also been linked to epigenetic modifications and neuroimmune activation, including altered DNA methylation and microglial-mediated inflammation, which can disrupt synaptic pruning and contribute to ASD-related phenotypes [48]. In addition, maternal or fetal hormonal perturbations caused by pharmaceuticals or environmental EDCs suggest epigenetic mechanisms with potential transgenerational effects [49]. Despite these findings, inconsistencies remain. A systematic review of 27 studies reported no consistent association between prenatal EDC exposure and autistic traits, highlighting limitations such as small sample sizes, insufficient mixture modeling, and inadequate sex-specific analyses [50]. Current evidence suggested that EDCs can affect neurodevelopment and behavior through hormonal, epigenetic, and neuroimmune mechanisms. Among chemical classes, polybrominated diphenyl ethers (PBDEs) have shown some of the most consistent associations, with prenatal exposure repeatedly linked to lower IQ, impaired reading ability, and increased externalizing behaviors in children [51]. Despite some uncertainties, these findings reinforced their role as key neurotoxicants in early life and underscored the need for longitudinal studies integrating mechanistic endpoints.

## Research gaps and future perspectives

Despite growing interest in EDCs and child health, significant gaps remain. Current biomonitoring methods are limited in detecting complex mixtures, especially during critical developmental periods. More standardized and sensitive techniques are needed for accurate exposure assessment. Promising approaches include mixture-oriented statistical methods such as Bayesian kernel machine regression and weighted quantile sum regression, which can better reflect real-world co-exposures. Further research is required using animal models, epidemiological studies, and computational tools to clarify long-term effects. Data on exposure during pregnancy, birth, and early life are still scarce, particularly in low- and middle-income countries (LMICs), where systematic biomonitoring is limited. Special attention should be given to high-risk populations and critical windows of susceptibility, such as fetal development, the neonatal period, and early childhood. Future studies should adopt longitudinal designs, advanced biomarkers, and multi-omics approaches to uncover underlying mechanisms and identify vulnerable populations. At the same time, stronger regulatory frameworks are needed. Policy recommendations should be tailored to regional contexts: in high-income countries, priority should be given to revising regulatory thresholds to account for chemical mixtures; in LMICs, low-cost biomonitoring programs and integration of EDC surveillance into maternal and child health services are urgently needed. Better integration of science into policy is essential to reduce EDC exposure and protect children’s health.

## Strengths and limitations

This study represented the first comprehensive and systematic bibliometric analysis of publications on EDCs and children’s health, offering an intuitive and objective overview that serves as a valuable reference for clinicians and researchers. Multiple bibliometric tools were applied to explore key research topics from diverse perspectives. Nevertheless, several limitations should be acknowledged: (1) The literature search was finalized on 4 June 2025, which may have excluded some recently published influential studies. (2) The search was confined to articles and reviews indexed in WoSCC to ensure high-quality sources, thereby excluding other publication types like books, case reports, clinical trials, and meta-analyses that might contain pertinent information. (3) The reliance on WoSCC meant that studies indexed exclusively in other major databases, such as PubMed, Embase, and Ovid, were not included, which represents a notable limitation, particularly given that many studies were conducted in the US. (4) Only publications in English were considered, potentially overlooking significant research published in other languages.

## Conclusions

This study presented a comprehensive bibliometric analysis of global research on EDCs and children’s health from 2005 to 2025. Through systematic mapping and visualization, we identified key contributors, research trends, and emerging hotspots in the field. The US played a leading role in both research output and influence, while China showed rapid growth in publication volume. Current hotspots focused on early-life exposure pathways, endocrine-related developmental disorders such as precocious puberty and thyroid dysfunction, and neurodevelopmental and behavioral outcomes. Research on EDC mixtures and their long-term impacts was gaining momentum, reflecting growing public health concerns. In summary, this study offers valuable insights into the evolution of EDC-related pediatric research and highlights the need for improved exposure assessment, mechanism-based studies, and stronger science-policy integration to protect future generations.

## Supplementary Material

Supplementary Table S1.docx

## Data Availability

The data for this study were obtained from the Web of Science Core Collection, a publicly accessible database. This bibliometric analysis focused on publications from 1 January 2005 to 4 June 2025 related to EDCs and children’s health. The datasets generated and analyzed during the study are available from the corresponding author upon reasonable request. Additional information or data supporting the study’s findings can also be requested from the corresponding author.
