# Supplementary material for: Bibliometric and visual analysis of global research on endocrine-disrupting chemicals and children’s health: evidence, emerging concerns, and research gaps
Source: Glob Health Action. 2025 Oct 16;18(1):2572014. doi: 10.1080/16549716.2025.2572014 (PMC12532360; doi:10.1080/16549716.2025.2572014)
Supplement: Supplementary Table S1.docx [file ZGHA_A_2572014_SM8346.docx]

***Supplementary Table S1. Software and parameter settings for bibliometric analyses***

| ***Software/***  ***Package*** | ***Analysis type*** | ***Key parameter settings*** |
| --- | --- | --- |
| *VOSviewer (v1.6.19)* | *Keyword co-occurrence, co-authorship, co-citation* | *Minimum keyword co-occurrence threshold = 20; normalization = association strength; clustering algorithm =modularity-based; minimum cluster size = 5; counting method = fractional counting* |
| *CiteSpace (v6.3.R1, 64-bit Advanced)* | *Keyword co-occurrence, keyword clustering, co-citation (journal/author/reference), timeline and timezone views, country/institution networks* | *Timespan = 2005–2025; slice length = 1 year; selection criteria = g-index (k = 10 or 25 depending on analysis); LRF = 3.0; L/N = 10; LBY = 5; e = 1.0; clustering algorithm = LLR; pruning = MST; network density = 0.004–0.091; Modularity Q = 0.40–0.75; silhouette = 0.72–0.90* |
| *Bibliometrix (R package v4.3.1)* | *Productivity analysis, co-authorship, country and institution performance, H-index statistics* | *Functions: biblioAnalysis() for descriptive statistics; biblioNetwork() for co-authorship and co-citation matrices; counting method = fractional counting; time window = 2005–2025* |
